# Neuroendocrine Tumor, Well Differentiated, of the Breast: A Relatively High-Grade Case in the Histological Subtype

**DOI:** 10.1155/2013/204065

**Published:** 2013-04-30

**Authors:** Shogo Tajima, Hajime Horiuchi

**Affiliations:** ^1^Division of Pathology, Shizuoka Saiseikai General Hospital, Shizuoka, Japan; ^2^Division of Pathology and Laboratory Medicine, NTT Medical Center, Tokyo, Japan

## Abstract

Primary neuroendocrine carcinoma of the breast is a rare entity, comprising <1% of breast carcinomas. Described here is the case of a 78-year-old woman who developed an invasive tumor in the left breast measuring 2.0 cm x 1.5 cm x 1.2 cm. The tumor was composed of only endocrine elements in the invasive part. It infiltrated in a nested fashion with no tubular formation. Intraductal components were present both inside and outside of the invasive portion. Almost all carcinoma cells consisting of invasive and intraductal parts were positive for synaptophysin and neuron-specific enolase. According to the World Health Organization classification 2012, this tumor was subclassified as neuroendocrine tumor, well-differentiated. Among the subgroup, this tumor was relatively high-grade because it was grade 3 tumor with a few mitotic figures. Vascular and lymphatic permeation and lymph node metastases were noted. In the lymph nodes, the morphology of the tumor was similar to the primary site. No distant metastasis and no relapse was seen for one year after surgery. The prognosis of neuroendocrine carcinomas is thought to be worse than invasive mammary carcinomas, not otherwise specified. Therefore, immunohistochemistry for neuroendocrine markers is important in the routine practice to prevent overlooking neuroendocrine carcinomas.

## 1. Introduction

Although some invasive ductal carcinomas of the breast show areas of neuroendocrine differentiation, primary neuroendocrine carcinoma of the breast is a rare entity, comprising <1% of breast carcinomas. In 2003, the World Health Organization (WHO) recognized this category and defined mammary neuroendocrine carcinoma as expression of neuroendocrine markers in more than 50% of tumor cells. In 2012, WHO revised the category and divided neuroendocrine carcinomas into three subtypes: neuroendocrine tumor, well-differentiated; neuroendocrine carcinoma, poorly differentiated/small cell carcinoma; and invasive breast carcinoma with neuroendocrine differentiation [1]. The presence of intraductal components is strong evidence for certifying the origin of a carcinoma as the breast. We present here a case of a neuroendocrine tumor, well-differentiated, of the primary breast origin showing only endocrine elements and no exocrine elements in the invasive part. 

## 2. Case Report

A 78-year-old woman was referred to NTT Medical Center Tokyo with a mass in the left breast that was detected by mammography during breast cancer screening. Levels of tumor markers such as carcoinembryonic antigen (CEA) and cancer antigen 15-3 (CA15-3) were found to be normal (2.5 ng/mL and 6.9 U/mL, resp.). Computer tomography showed a well-circumscribed mass (1.5 × 0.9 cm) and a swollen lymph node (1.0 × 0.8 cm). No other tumors were detected in any other organs. Aspiration cytology was performed and the mass was diagnosed as a carcinoma. The patient was subsequently admitted to the Medical Center for surgery. She underwent a partial mastectomy and level I lymphadenectomy without sentinel lymph node identification. 

 Anastrozole was administered postoperatively. The serum tumor marker NSE showed no remarkable change for one year after surgery. No tumoral mass was seen on the computer tomography images.

## 3. Pathological Findings

### 3.1. Gross Examination

The surgical specimen (15.5 × 12.0 × 4.0 cm) contained a grayish-white tumor measuring 2.0 × 1.5 × 1.2 cm with a well-demarcated border. 

### 3.2. Microscopic Examination

**Figure 1:**
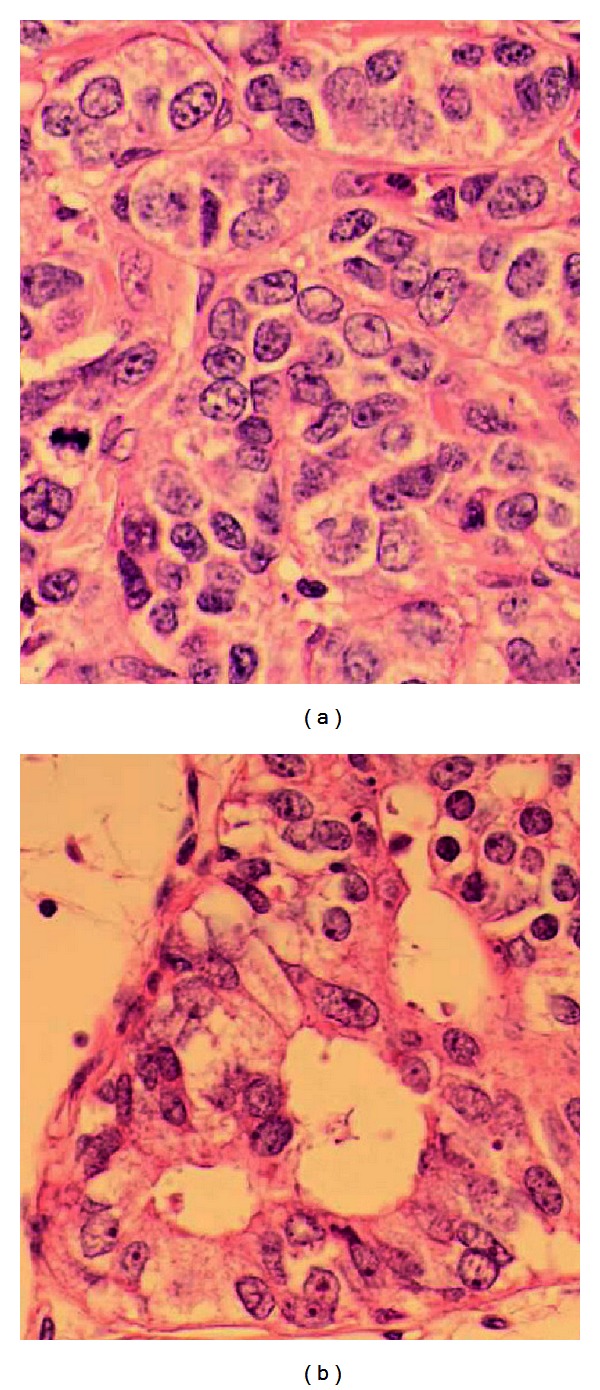
(a) Nested pattern of growth is shown. A mitotic figure can be observed. (H&E, 40×). (b) Intraductal component (H&E, 40×).

The tumor was an invasive carcinoma with a minor intraductal component. The invasive component was composed of carcinoma cells displaying nested pattern of growth with no tubular formation (Figure 1(a)). Nuclear pleomorphism was moderate. Mitotic figures were relatively prominent (18 figures per 10 high-power fields at the highest area; field diameter 0.50 mm). Considering these factors, the tumor was grade 3 [2]. Necrosis was scarce, although several apoptotic cells were observed. The intraductal components were present both inside and outside of the invasive area. Columnar-shaped tumor cells were only detected in the intraductal component (Figure 1(b)). Vascular permeation and lymphatic permeation were also seen. Lymph node (level I) metastases were confirmed in 6 out of 10 samples and all of them were macrometastases. 

**Figure 2:**
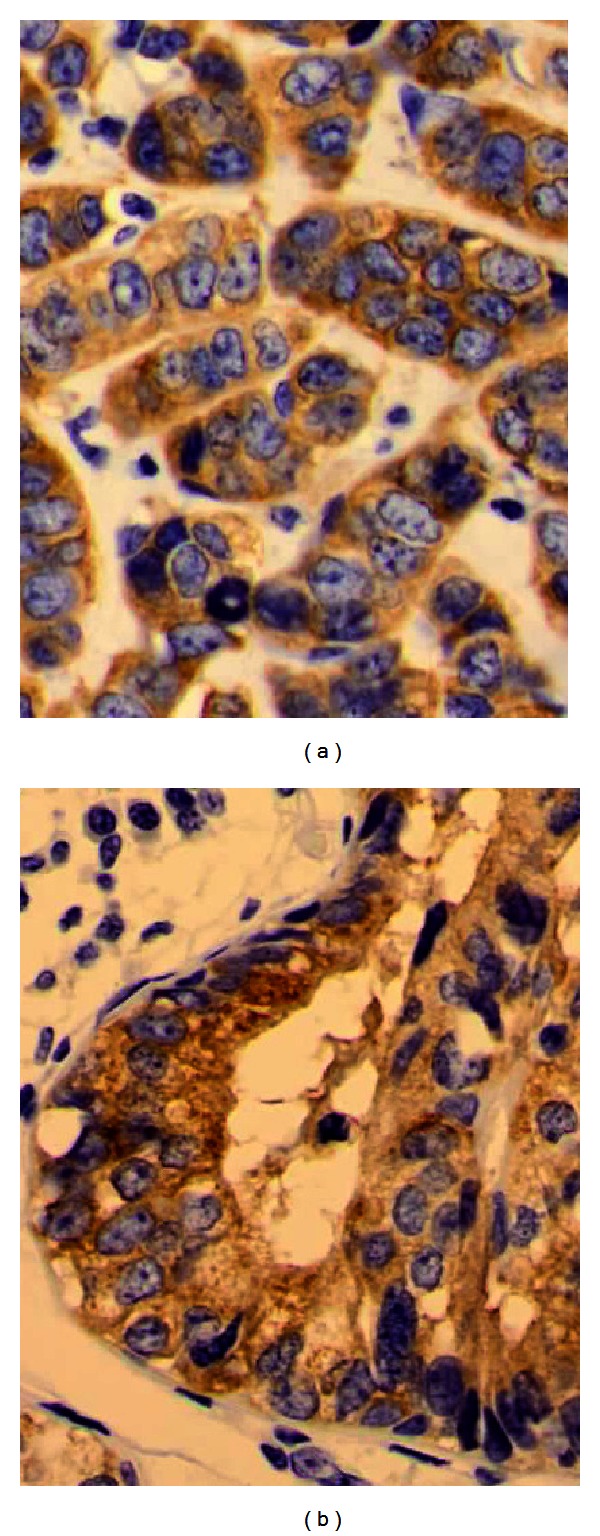
(a) Almost all the cells are positive for synaptophysin (40×). (b) Intraductal component (40×).

Immunohistochemical staining was positive for synaptophysin (Figures 2(a) and 2(b)), NSE, CK7, and ER in almost all the tumor cells; however, PgR was positive only focally. Immunohistochemical staining for chromogranin A and NCAM was negative. Ki67 labeling index was about 32% at the highest area (counting 1,000 cells). The intraductal components were highlighted by p63-positive myoepithelial cells. In situ hybridization revealed no significant *Her2*/*neu *gene amplification. 

## 4. Discussion

In 2012, WHO divided carcinomas with neuroendocrine features into three categories: neuroendocrine tumor, well-differentiated; neuroendocrine carcinoma, poorly differentiated/small cell carcinoma; and invasive breast carcinoma with neuroendocrine differentiation [1]. This case is compatible with the definition of a neuroendocrine tumor, well-differentiated. 

The neuroendocrine markers synaptophysin and neuron-specific enolase were found to be positive in almost all the tumor cells. Immunohistochemical positivity to CK7 and ER supports the diagnosis of primary breast tumor, and the presence of an intraductal component with peripheral p63-positive myoepithelial cells is also strong evidence of the breast as the tissue of origin.

Majority of the tumors under the category of neuroendocrine tumor, well-differentiated, are known to be of low or intermediate grade [1]. However, the tumor of the present case was grade 3, and Ki67 labeling index (32%) is so high that this case is considered to be of relatively high grade in the subgroup. It was reflected in the fact that vascular and lymphatic permeation and also lymph node metastases was seen. No distant metastasis and no relapse were seen for one year after surgery.

 The prognosis of neuroendocrine carcinoma is thought to be worse than invasive mammary carcinoma, not otherwise specified [3, 4]. Therefore, the distinction between them is very important. Growth patterns occurring in neuroendocrine carcinomas are solid, alveolar, and nested [1]. Neuroendocrine markers are usually not routinely stained, so there might be some cases that are overlooked. It is wise to stain them when solid, alveolar, or nested pattern of growth is prominent. 

## 5. Conclusion

This report describes a neuroendocrine tumor, well-differentiated, that shows relatively high-grade morphological and biological features. It is recommended to differentiate neuroendocrine carcinomas from other invasive mammary carcinomas by routinely using immunohistochemistry when solid, alveolar, or nested pattern of growth is prominent because the former is thought to show worse prognosis [3, 4].
